# Crosstalks Between Gut Microbiota and *Vibrio Cholerae*

**DOI:** 10.3389/fcimb.2020.582554

**Published:** 2020-10-23

**Authors:** Zixin Qin, Xiaoman Yang, Guozhong Chen, Chaiwoo Park, Zhi Liu

**Affiliations:** Department of Biotechnology, College of Life Science and Technology, Huazhong University of Science and Technology, Wuhan, China

**Keywords:** gut microbiota, *Vibrio cholerae*, T6SS, QS, ROS, pH, Bioactive metabolites

## Abstract

*Vibrio cholerae*, the causative agent of cholera, could proliferate in aquatic environment and infect humans through contaminated food and water. Enormous microorganisms residing in human gastrointestinal tract establish a special microecological system, which immediately responds to the invasion of *V. cholerae*, through “colonization resistance” mechanisms, such as antimicrobial peptide production, nutrients competition, and intestinal barrier maintenances. Meanwhile, *V. cholerae* could quickly sense those signals and modulate the expression of relevant genes to circumvent those stresses during infection, leading to successful colonization on the surface of small intestinal epithelial cells. In this review, we summarized the crosstalks profiles between gut microbiota and *V. cholerae* in the terms of Type VI Secretion System (T6SS), Quorum Sensing (QS), Reactive Oxygen Species (ROS)/pH stress, and Bioactive metabolites. These mechanisms can also be applied to molecular bacterial pathogenesis of other pathogens in host.

## Introduction

To date, the devastating diarrheal disease cholera pandemics have been occurred seven times, and it is still endemics in the world, responsible for up to 3 million cases and 100,000 deaths annually (Theriot and Petri, 2020). *Vibrio cholerae* is the causal organism of the disease cholera, usually infects humans through ingestion of contaminated water and food, colonizes on the surface of small intestine villi with the aid of toxin coregulated pilus (TCP), and then secretes cholera toxin (CT), causing watery diarrhea and vomiting that lead to severe dehydration and even death (Yoon and Waters, 2019). *V. cholerae* is gram-negative, curved and facultative bacterium, with a long unipolar flagellum. According to the serological characteristics of its surface O-antigens, *V. cholerae* has over 200 serotypes, in which only O1 serotype cause the cholera pandemics. Based on the genotypes, O1 *V. cholerae* are further classified into classical biotype and El Tor type (Kaper et al., 1995). Classical biotype was the causative agent of the first six cholera epidemics, while El Tor *V. cholerae* caused the seventh epidemic (Albert, 1994).

In 1894, Metchnikoff, the Russian Nobel laureate, claimed that cholera was a disease to humans due to the fact that the phenotype of human infections could not be precisely replicated in the infections of laboratory model animals (Ritchie and Waldor, 2009). He further speculated that experimental animals could not be infected with *V. cholerae* because of the presence of microorganisms in the gut and suggested that animal cubs could be used as an experimental model because of their significantly lower abundance of gut microbes. Indeed, the 3–5-day-old infant mouse is used as the most common model for *V. cholerae* pathogenesis research. However, there are enormous bacteria resided in host gastrointestinal environment, with the 10 times more population than those of host cell, those gut microbes provide “colonization resistance” against pathogen invasion. Gut microbiota can produce short-chain fatty acids, antibacterial substances, signal molecules, and bioactive metabolites to benefit host health and to defend pathogenic bacterial infection, such as *V. cholerae* (Ducarmon et al., 2019). Correspondingly, *V. cholerae* can also sense the change of environments and adjust gene expression to increase adaptability (Parker and Sperandio, 2009).

In recent years, an increasing number of researchers turned to study *V. cholerae* pathogenesis under the microbiota background. This review will elaborate the crosstalk profiles between gut microbiota and *V. cholerae* and reveal their synergistic and antagonistic effects from the following aspects: (1) Type VI Secretion System (T6SS), (2) Quorum Sensing (QS), (3) Reactive Oxygen Species (ROS) and pH, (4) Bioactive metabolites. In this review, we aspire to shine the light on the gut microbiota modulation as a promising therapy for *V. cholerae* and related enteric pathogens infection.

## T6SS-Depended Crosstalk Between Gut Microbiota and *V. Cholerae*

**Figure 1:**
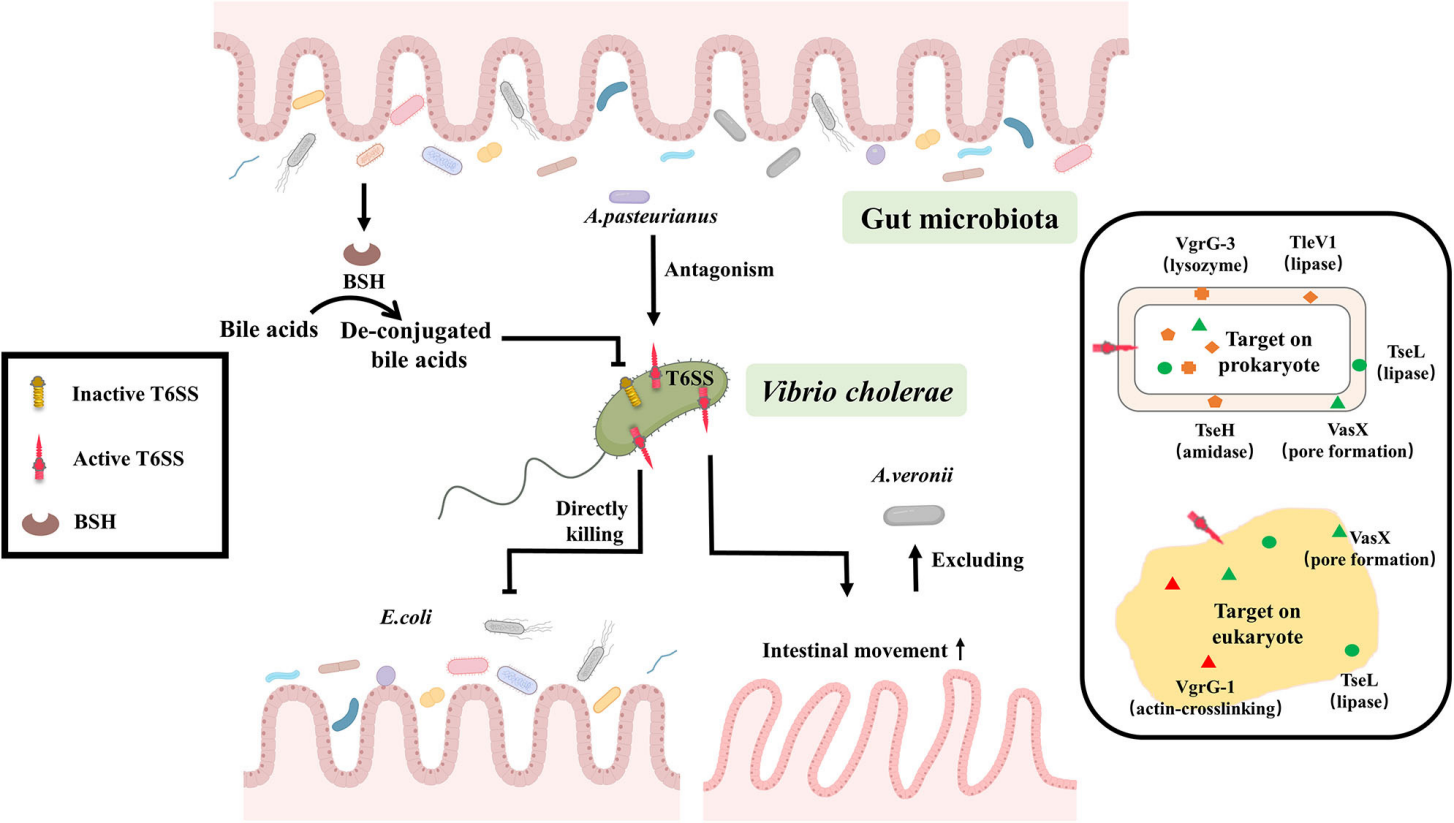
T6SS-depended crosstalk between gut microbiota and *V. cholerae*. Gut microbiota can suppress the expression of *V. cholerae* T6SS by converting bile acids to de-conjugated bile acids through the production of BSH, or some gut bacteria like *A. pasteurianus* can enhance the virulence of *V. cholerae* through antagonizing with *V. cholerae* T6SS. Conversely, *V. cholerae* T6SS has direct lethal effects on the prokaryote, such as *Escherichia coli*. Or it can stimulate the peristalsis of intestinal epithelial cells for excluding gut microbiota, like *Aeromonas veronii*, to improve the efficiency of the colonization. The effector of T6SS plays an important role in those reactions. Six T6SS effectors with corresponding functions were reported in *V. cholerae*, among them, TleV1 (lipase), TseH (amidase), the C-terminal domain of VgrG-3 (lysozyme) targeted to prokaryotic cells, the C-terminal domain of VgrG-1 (actin-crosslinking) attacked eukaryotes, and VasX (pore formation), TseL (lipase) acted on both prokaryotic and eukaryotic cells.

T6SS is a syringe-like protein apparatus, affects the physiological function of susceptible cells by injecting toxic effectors into the cells, including prokaryotes as well as eukaryotes, and even lysing susceptible cells (Pukatzki et al., 2006; Schwarz et al., 2010). Up to 25% of Gram-negative bacteria, including *V. cholerae*, have been reported to contain T6SS (Pukatzki et al., 2006; Bingle et al., 2008; Basler et al., 2012), The *V. cholerae* T6SS consists of the following components: substrate proteins (HisF, VasA, VasB, VasE, and VasJ) attached to the cell outer membrane via protein-to-protein linkages, secretion-promoting tubular sheath exoskeleton protein VipA/VipB (Basler et al., 2012; Broms et al., 2013), sheath protein-coated a tubule of hemolysin co-regulated proteins (Hcps), VgrG protein responsible for punching holes in receptor cells and effector toxin (Shneider et al., 2013; Cianfanelli et al., 2016). Until now, six T6SS effectors with corresponding functions were reported in *V. cholerae*, among them, TleV1 (lipase), TseH (amidase), the C-terminal domain of VgrG-3 (lysozyme) targeted to prokaryotic cells, the C-terminal domain of VgrG-1 (actin-crosslinking) attacked eukaryotes, and VasX (pore formation), TseL (lipase) acted on both prokaryotic and eukaryotic cells (Pukatzki et al., 2007; Miyata et al., 2011; Russell et al., 2011, 2013). Function of *V. cholerae* T6SS only performed when its VipA/VipB protein was contracted rather than extended (Figure 1). Susceptible strains can be attacked by T6SS with its toxic effectors, so they have evolved a number of protective mechanisms, such as the production of cognate immune proteins or extracellular polysaccharides (Dong et al., 2013; Fu et al., 2013; Toska et al., 2018). The protective mechanisms, while remaining to be further excavated, may explain why some gut microbes are less vulnerable to T6SS attacks (Fast et al., 2018). Overall, T6SS plays an important role in the interaction between gut microbiota and *V. cholerae*.

Gut microbiota can suppress the expression of *V. cholerae* T6SS by converting bile acids to de-conjugated bile acids through the production of bile salt hydrolase (BSH) (Bachmann et al., 2015; Alavi et al., 2020). While the antagonistic interaction of intestinal microbes with *V. cholerae* T6SS enhances the virulence of *V. cholerae* either in mouse (Zhao et al., 2018), rabbit (Fu et al., 2018) or *Drosophila* model (Fast et al., 2018, 2020), concomitantly, the regenerative function and cell differentiation of intestinal cell are inhibited, resulting in massive intestinal cells shedding and then the exacerbation of the cholera symptoms (Fast et al., 2018, 2020). However, the precise mechanism of interaction between gut microbiota and *V. cholerae* by T6SS is largely unknown and needs to be further explored.

Conversely, *V. cholerae* can use T6SS to enhance its own adaptation in the gut. *V. cholerae* T6SS has direct lethal effect on the Prokaryotic organisms such as *Escherichia coli* MG1655, *Pseudomonas aeruginosa* (Basler et al., 2012, 2013; Dong et al., 2013; Fu et al., 2013; Fast et al., 2018). It can also stimulate the peristalsis of intestinal epithelial cells for excluding gut microbiota, like *Aeromonas veronii*, to improve the efficiency of the colonization (Logan et al., 2018; Booth and Smith, 2020). This suggests that T6SS is a critical method of communication between gut microbiota and *V. cholerae* (Figure 1).

## QS-Depended Crosstalk Between Gut Microbiota and *V. Cholerae*

QS is widely present in different bacteria and adjusts the behavioral changes of the entire population according to the cell density. Different gut microbiota produces different quorum sensing molecules. For example, Acyl-homoserine lactone (AHL) as autoinducer molecule is produced by gram-negative bacteria. Small-molecule polypeptides are produced by gram-positive bacteria. Both types of bacteria can produce the furanyl dibasic compound (2S, 4S)-2-methyl-2,3,3,4-tetrahydroxytetrahydrofuran borate (AI-2). Intestinal microbiota also communicates with each other via quorum sensing signals, transforming intestinal pathogenic commensal bacteria into pathogenic bacteria, and thus impacts on host health (Kim et al., 2020). While *V. cholerae* itself can produce three QS signal molecules, including inter-species communication autoinducer AI-2 (Schauder et al., 2001; Chen et al., 2002), intra-genus-specific autoinducer (S)-3-hydroxytridecan-4-one (CAI-1) (Kelly et al., 2009) and 3,5-dimethylpyrazin-2-ol (DPO) (Herzog et al., 2019). *V. cholerae* adapts to the gut environment through QS signals either by influencing the expression of the primary regulator *hapR*, or through the *vqmA* pathway, which regulates a number of physiological pathways, such as the expression of virulence factors, biofilms, T6SS, the formation of natural transformation states, cell aggregation, and other behaviors (Miller et al., 2002; Zhu et al., 2002; Hammer and Bassler, 2003; Beyhan et al., 2007; Shikuma et al., 2009; Suckow et al., 2011; Lo Scrudato and Blokesch, 2012; Shao and Bassler, 2014; Hawver et al., 2016; Jemielita et al., 2018). It is not surprised that QS is important for communication between gut microbiota and *V. cholerae*.

Gut microbiota can influence the physiological status of *V. cholerae* through QS. Ansel Hsiao found that the abundance of *Ruminococcus obeum* was significantly increased in the gut microbiota involved in recovery from *V. cholerae* infection. Further analysis of the function of *R. obeum* on *V. cholerae* revealed that the AI-2 signal produced by *R. obeum* significantly enhanced the colonization of *V. cholerae*. Interestingly, the AI-2 synthesized by *R. obeum luxS* does not act through the *V. cholerae* AI-2 sensor, LuxP, but rather affects the expression of *V. cholerae* virulence through another pathway that involves high expression of *vqmA* (Hsiao et al., 2014). Some researchers speculated that this phenotype may be related to the accumulation of signal molecule DPO, which can activate the QS system via *vqmA* pathway, but the actual mechanism is controversial (Papenfort et al., 2017). Also, recently reported intestinal metabolite ethanolamine, is recognized by CqsR and then up-regulate the expression of *hapR* (Watve et al., 2020), can activate the QS system of *V. cholerae*. It shows that the QS signals produced by gut microbiota are complex and diverse, and it remains to be discovered if there is any communication of other group-sensing signals between gut microbiota and *V. cholerae*.

On the contrary, QS signals produced by *V. cholerae* may also have an effect on the physiological function of other microorganisms. For example, the CAI-1 produced by *V. cholerae* enhances the expression of T3SS virulence of enteropathogenic *Escherichia coli* (EPEC) E2348/69 and subsequent diarrhea in the host (Gorelik et al., 2019). Also, the *V. cholerae* QS system is still poorly understood, so there may be other factors by which *V. cholerae* communicates with gut microbiota via QS signals.

**Figure 2:**
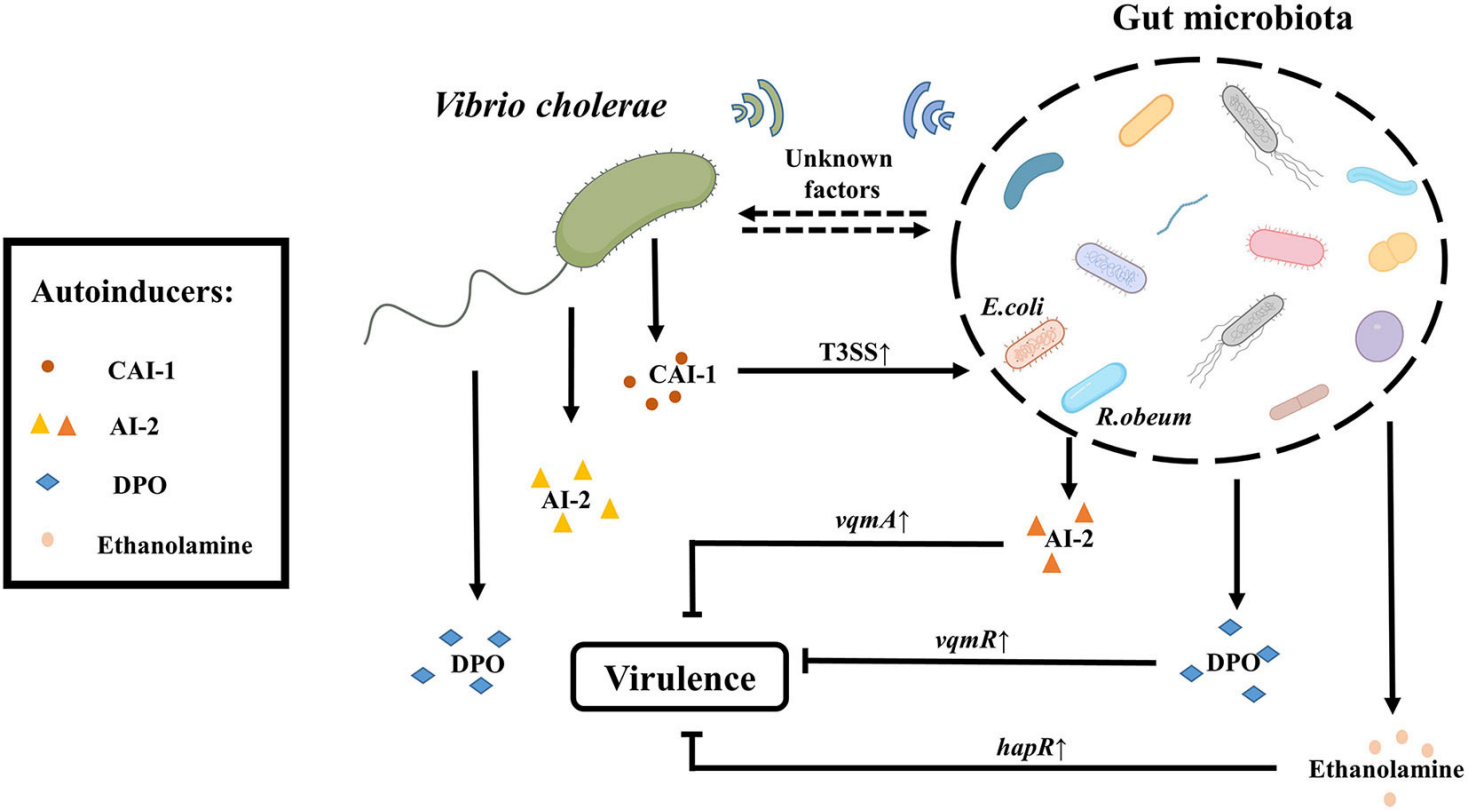
QS- depended crosstalk between gut microbiota and *V. cholerae*. The gut microbiota like *R. obeum* can reduce the colonization of *V. cholerae* in the intestine by generating the quorum sensing signal AI-2 (different from the *V. cholerae* AI-2). The gut microbiota can also suppress the virulence of *V. cholerae* through metabolites such as ethanolamine or DPO mediated by *hapR* and *vqmR*, respectively. In addition, there are a series of unknown signaling molecules to be exploited. Conversely, *V. cholerae* itself can produce three kinds of autoinducers including CAI-1, AI-2, and DPO. CAI-1 produced by *V. cholerae* can enhance the pathogenicity of *E. coli* by up-regulating the expression of T3SS-associated genes. And there may be other factors by which *V. cholerae* communicates with gut microbiota via QS signals.

Because QS plays a critical role in interbacterial communication, the combination of QS signaling and synthetic biology methods to modify intestinal probiotics for detection, prevention, and treatment of *V. cholerae* infections has become a new therapeutic approach. For example, modified *Escherichia coli* Nissle1917 can produce *V. cholerae*-derived CAI-1 to reduce colonization of *V. cholerae* (Duan and March, 2008, 2010), or engineered *Lactococcus* lactis subsp. cremoris MG1363 can detect *V. cholerae* in the fecal through CAI-1 signals which is applied for the readily detection of *V. cholerae* (Higgins et al., 2007; Holowko et al., 2016; Mao et al., 2018). In summary, the *V. cholerae* QS will perform different functions depending on various environmental conditions. For *V. cholerae*, either external addition of CAI-1 chemicals (Higgins et al., 2007) or overexpression of CAI-1 using probiotics as carriers (Duan and March, 2008, 2010) inhibits the colonization of *V. cholerae*, whereas for other intestinal microorganisms, such as EPEC, CAI-1 activates their T3SS expression to enhance virulence. This also reflects the complexity and diversity of the gut environment. Therefore, better understanding of the *V. cholerae* QS system can provide a solid theoretical basis for the clinical treatment for cholera. It is becoming an increasingly promising therapy to mitigate and prevent the *V. cholerae* infection via interfering with the QS signal (Figure 2).

## ROS/pH-Depended Crosstalk Between Gut Microbiota and *V. Cholerae*

Pathogens invading the intestinal environment primarily challenge intestinal innate immunity, which includes ROS and low pH. It is reported that the diversity of the gut microbiota is related to the level of ROS (Yardeni et al., 2019). Gut microbiota can produce ROS by itself (Chen et al., 2019). Meanwhile, L-lactate produced by *Lactobacillus* plantarum can either mediate the consumption of NADH by NOX enzymes or be metabolized in mitochondria through the generation of pyruvate, enhancing ROS levels in the intestine (Iatsenko et al., 2018). Whereas, gut microbiota can also reduce ROS production by metabolizing Short-chain fatty acids (SCFAs) such as N-butyrate (Mottawea et al., 2016) or scavenge ROS by peroxidase (Yoon et al., 2016) to diminish ROS levels in the gut.

To better adapt to the intestinal environment, *V. cholerae* has also evolved a series of mechanisms to defend against or scavenge ROS, including the production of antioxidant molecule like glutathione (Meister and Anderson, 1983), catalase like KatB/KatG (Xia et al., 2017), superoxide dismutase like OhrA/AphC (Cha et al., 2004; Liu et al., 2011, 2016; Wang et al., 2017), even it can protect itself from damaging by high levels of ROS in the intestinal environment through transforming its morphology, such as reversible phase variation between the rugose and smooth colony variants to response to ROS or biofilm formation (Faruque et al., 2006; Sengupta et al., 2016; Wang et al., 2018). Conversely, under inflammatory conditions *V. cholerae* impacts the structure and composition of intestinal microorganisms in multiple pathways. *V. cholerae* can produce a cholera toxin (CT), which causes electrolyte imbalance in the intestine, leading to diarrhea and thus disrupts the structure of intestinal commensal bacteria. Besides, under inflammatory conditions, the concentrations of ${\text{NO}}_{3}^{-}$ increases and the available iron concentration decreases, while *V. cholerae* can make better use of ${\text{NO}}_{3}^{-}$ as a receptor for the electron respiratory chain and thus multiply faster and occupy an ecological niche (Bueno et al., 2018). *V. cholerae* also could promote their own proliferation by competing with gut microbiota and the host with iron (Rivera-Chavez and Mekalanos, 2019).

Apart from ROS, the host gut environment also has low pH pressure. pH is an important factor for bacterial growth in the gut. Gut microbiota can also alter pH to resist the invasion of *V. cholerae*. Culture supernatants of *Lactobacillus lactis* isolated from feces of healthy children inhibited the biofilm formation of *V. cholerae*. The phenotype that inhibited biofilm formation largely vanished after neutralization of the culture supernatant (Kaur et al., 2018). In addition, *Escherichia coli* 40 and Nissle 1917 isolated from the gut of healthy human volunteers were co-cultured with *V. cholerae* N16961 in LB medium containing glucose, respectively. It was found that both of them reduced the pH value in the medium and affected on the survival rate of *V. cholerae* (Sengupta et al., 2017). In the zebrafish model, the glucose combination with *Escherichia coli* 40 or Nissle 1917 reduced the colonization of *V. cholerae* N16961 by changing the pH in the gut (Nag et al., 2018). This is consistent with the use of glucose-based oral rehydration (ORS) combination with probiotic *Escherichia coli* during cholera treatment. These suggest that gut microbiota and their compositions can inhibit the colonization of *V. cholerae* by changing pH.

Low pH in the intestinal microenvironment affects colonization of *V. cholerae*, and the reason may be that *V. cholerae* can respond to low pH by multiple mechanisms. For example, under hypoxic growth condition, *V. cholerae* can use nitrate as an oxidative phosphorylation electron acceptor, and adjust the process of nitrate/nitrite according to the environmental pH, affecting its own adaptability by inhibiting glycolysis and proton motive force (PMF) (Bueno et al., 2018). In addition, NhaP1, as an antiporter of K^+^(Na^+^)/H^+^, enables *V. cholerae* to grow under low pH condition and maintain internal pH homeostasis by removing K^+^/Na^+^ from the cytoplasm and ingesting H^+^. The H^+^ enters the respiratory chain and is consumed. This mechanism is more suitable for *V. cholerae* to adapt intestinal microenvironment (Quinn et al., 2012). *V. cholerae* can also regulate lysine decarboxylase by AphB to consume H^+^ so that it can alleviate low pH states (Kovacikova et al., 2010). Under alkaline conditions, *V. cholerae* can suppress related acid tolerance genes via OmpR and increase fitness (Kunkle et al., 2020). Indeed, *V. cholerae* itself has different pH patterns of fermentation depended on glucose. El Tor N16961 can produce 2, 3-butanediol as the neutral product of fermentation, avoiding the acidification of the medium. In contrast, classic biotype O395 is unable to synthesize 2, 3-butanediol, therefore its viability is diminished during mixed fermentation with glucose due to the acidification of the medium by synthetic organic acids (Lee et al., 2020).

The mechanism by which *V. cholerae* affects gut microbiota by changing pH is unknown. However, *V. cholerae* can produce cholera toxin to cause intestinal inflammation of the host, resulting in intestinal electrolyte imbalance. This may affect pH changes to compete for niches.

**Figure 3:**
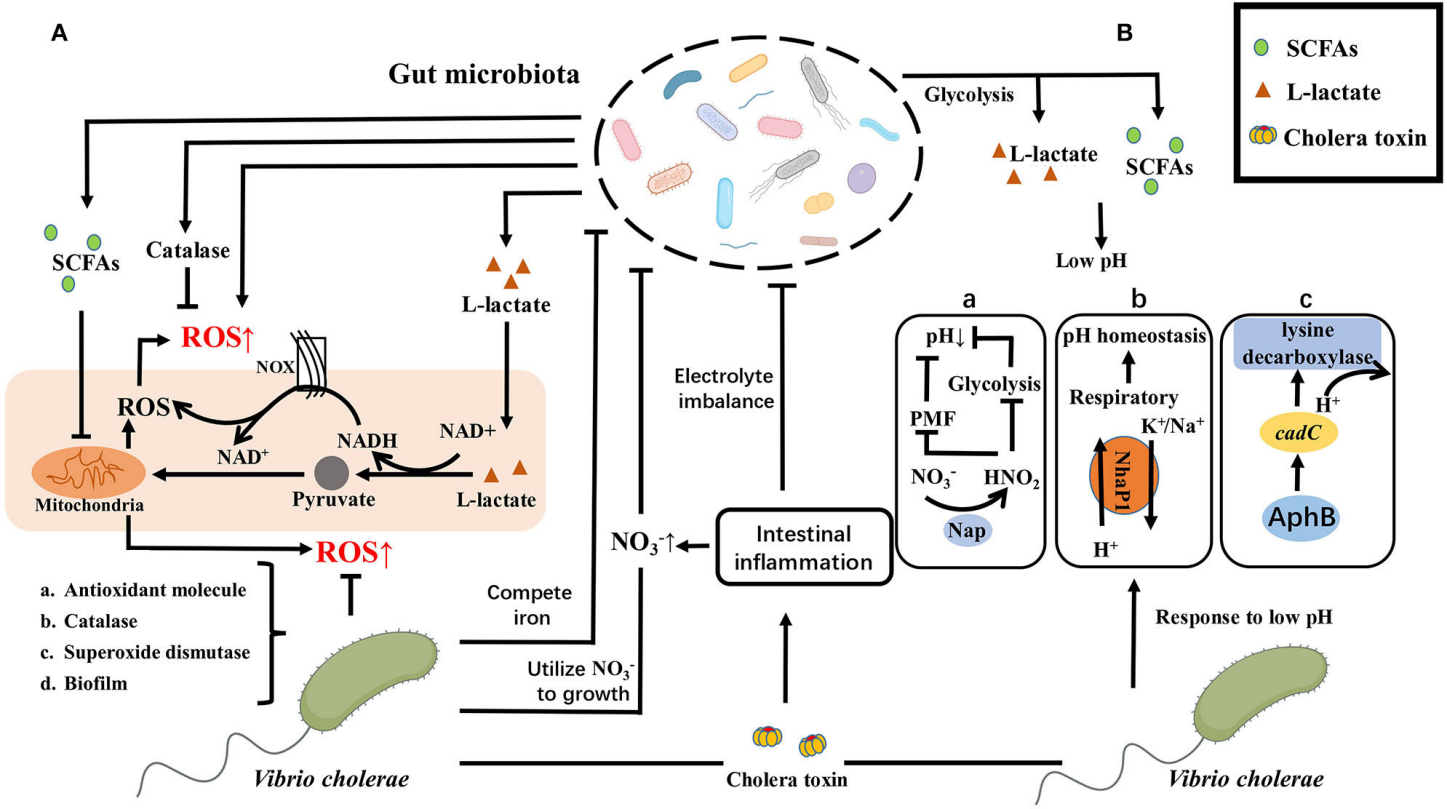
ROS/pH-depended crosstalk between gut microbiota and *V. cholerae*. **(A)** Gut microbiota can produce ROS directly or activate the host to produce ROS, such as L-lactate produced by *Lactobacillus Plantarum* can either mediate the consumption of NADH by NOX enzymes or metabolized in mitochondria through the generation of pyruvate, increasing ROS levels in the gut. While gut microbiota can also reduce ROS production by metabolizing SCFAs such as N-butyrate or scavenge ROS by peroxidase to diminish ROS levels in the gut. To better adapt to the intestinal microenvironment, *V. cholerae* has also evolved a series of mechanisms to defend against or scavenge ROS, including the production of (a) antioxidant molecule, (b) catalase, (c) superoxide dismutase, or (d) transform its morphology, like biofilm formation. Conversely, *V. cholerae* impacts the structure and composition of intestinal microorganisms in multiple pathways, including causing electrolyte imbalance in the intestine through the production of CT to disrupt the structure of intestinal commensal bacteria, or making better use of ${\text{NO}}_{3}^{-}$ as a receptor for the electron respiratory chain and competing with gut microbiota and the host with iron to promote their own proliferation and occupy an ecological niche. **(B)** *E. coli* glucose fermentation and *lactobacillus* metabolites create a low pH environment to prevent the *V. cholerae* from colonizing in the small intestine by producing SCFAs and L-Lactate. While *V. cholerae* has a variety of response strategies, containing (a) anaerobic nitrate respiration of *V. cholerae* on bacterial expansion dependent on pH by inhibiting glycolysis and PMF to reduce the acid formation; (b) the cytoplasmic K^+^/Na^+^ exchanged at low pH to maintain pH homeostasis; (c) the expression of *cadC* activated by AphB to consume H^+^. Same as ROS response, *V. cholerae* can also cause electrolyte imbalance in the intestine by producing CT to disrupt the structure of intestinal commensal bacteria.

Thus, it is known that gut microbiota can influence the ROS or pH of the gut environment to interfere with the colonization and infection of *V. cholerae*. In turn, *V. cholerae* can develop mechanisms to defend against it. This indicates that the interaction between gut microbiota and *V. cholerae* is complex and versatile (Figure 3).

## Bioactive Metabolites-Depended Crosstalk Between Gut Microbiota and *V. Cholerae*

Bioactive metabolites are also an important way for the gut microbiota to communicate with each other. The main bioactive metabolites include SCFAs, bacteriocins, and bile acids.

SCFAs are fatty acid fermentation products of microorganisms with indigestible polysaccharides as substrates. The concentration of SCFAs is relatively abundant in the proximal colon of the host. SCFAs are metabolites of gut microbiota and participate in the regulation of various host physiological process (Dalile et al., 2019). Surveillance of clinical samples of *V. cholerae* infection found that the content of SCFAs in the host decreased and the probiotic *Bifidobacterium* abundance decreased after *V. cholerae* infection. As the treatment progresses, the abundance of *Bifidobacterium* and SCFAs were return to normal levels (Monira et al., 2010). Besides, mice treated with clindamycin reduced the abundance of *Bacteroides* and the content of SCFAs to enhance the colonization ability of *V. cholerae* (You et al., 2019). All these indicate that probiotics in the host can antagonize the colonization of *V. cholerae* by secreting SCFAs. In the process of infecting the host, *V. cholerae* can also activate the transcription of acetyl-CoA synthase-1 (ACS-1) through the two-component system CrbRS, thereby regulating the conversion of *V. cholerae* acetate to deplete the acetate in the intestinal environment for protecting itself. The absence of this acetate will cause the host's insulin signal transduction pathway to be blocked and the accumulation of lipids, which will affect the host's health (Hang et al., 2014).

Antimicrobial peptide is a kind of peptide with antibacterial activity, which can be divided into two categories in the intestinal environment: host-derived antimicrobial peptides and microbial-derived antimicrobial peptides (also known as bacteriocins). The host-derived antimicrobial peptides are mainly produced by intestinal epithelial cells and Pan's cells, including defensins, cathelicidins, lysozymes, chemokines, etc. (Chung and Raffatellu, 2019). During *V. cholerae* infection, the expression of host-derived antimicrobial peptides is upregulated, including α-defensin (HD-5 and−6), β-defensin (hBD-1-4), cathelicidin (LL-37), etc. (Qadri et al., 2004; Shirin et al., 2011). Human α-defensin generally damages bacteria by disrupting cell membranes, while HD-6 defends against pathogenic bacteria by trapping microbes in the intestinal lumen (Chairatana and Nolan, 2017). Human β-defensin can also mediate membrane lysis to exert antimicrobial activity, as well as capture or kill bacteria by inducing self-nets and neutrophil extracellular traps (NETs) (Alvarez et al., 2018). In addition, cathelicidin can mediate membrane perturbation and induce ROS production to inhibit bacterial growth (Rowe-Magnus et al., 2019). *V. cholerae* has also evolved a response mechanism that uses the major virulence protein cholera toxin CT to activate several intracellular signaling pathways involving protein kinase A (PKA), ERK-MAPKinase, and Cox-2 to downregulate the transcription of AMPs with cAMP accumulation (Chakraborty et al., 2008). The antimicrobial peptides or bacteriocin derived from bacteria can be divided into gram-positive bacteriocin and gram-negative bacteriocin according to the type of bacteria. In the study of bacteriocin, Nisin produced by the genus streptococcus is relatively extensive (Hammami et al., 2013). The culture supernatant of *Pediococcus acidilacticii* QC38 isolated from food producing bacteriocin, showed inhibitory activity on the growth of *V. cholerae in vitro*, indicating that bacteriocins produced by probiotic QC38 had antagonistic effect on *V. cholerae* (Morales-Estrada et al., 2016). Previously, *Lactobacillus casei* OGM12 isolated from the food also showed inhibitory activity against *V. cholerae*, which produces bacteriocin casein A (Olasupo et al., 1995). Also, the bacteriocin produced by *Streptococcus lactis* 11451 showed inhibitory activity against *V. cholerae in vitro* (Spelhaug and Harlander, 1989). These indicate that the bacteriocin produced by probiotics has antibacterial activity against *V. cholerae*.

*V. cholerae* possesses its own antagonistic mechanism against this antimicrobial active peptide. Resistance-nodulation-division (RND) mutants are sensitive to antimicrobial peptides *in vitro* and showed colonization defects on infant mice experiments. These phenotypes indicate RND efflux pump is important for *V. cholerae* to resist the toxicity of antimicrobial peptides (Bina et al., 2008). Besides, the outer membrane vesicles (OMV) of *V. cholerae* also play a crucial role in this stress condition. In the presence of antimicrobial peptides, the OMV content secreted by *V. cholerae* does not change, while the structure is altered, including the two outer membrane proteins OmpV and OmpW, as well as the Bap1 protein. Research showed that Bap1 protein can be combined with OmpT protein on the surface of OMV and can also be used as a ligand to capture antibacterial peptides to achieve the protection of bacteria (Duperthuy et al., 2013). *V. cholerae* also has a periplasmic space protein SipA, which can interact with the outer membrane protein OmpA. After the antibacterial peptide enters the periplasmic space, it may be captured by SipA, and then interact with OmpA to transport out antimicrobial peptides (Saul-McBeth and Matson, 2019).

Bile acids are an important substance involved in the regulation of the hepatic-intestinal axis. Primary bile acids are produced from the body's liver using cholesterol as a substrate, followed by bile and discharged into the intestinal cavity, and then metabolized by the gut microbiota secondarily, in the terminal jejunum or ileum through the portal vein reabsorption back to the liver. In the human body, the primary bile acids produced by the liver mainly include cholic acid (CA) and chenodeoxycholic acid (CDCA), and then form conjugated bile acid (CBA) with taurine or glycine to enter the gallbladder and transport to small intestine (Ridlon et al., 2016). Most bile salts would be absorbed by the body from the terminal ileum, but some bile salts will be metabolized by gut microbiota to produce secondary bile salts (Wahlström et al., 2016). *V. cholerae* in the mature biofilm state is disrupted by taurocholate by altering the biofilm matrix and promoting biofilm disintegration (Hay and Zhu, 2015). Besides, taurocholate stimulates the formation of C207-C207 disulfide bonds between TcpP molecules, thereby activating the expression of the virulence gene *toxT* (Yang et al., 2013). In the case of mucin-activated *V. cholerae* T6SS expression, the taurine and glycine groups in the conjugated deoxycholic acid will enhance the killing effect of *V. cholerae* T6SS on intestinal commensal bacteria. Thus, while conjugated secondary bile acids inhibit *V. cholerae* biofilm formation, *V. cholerae* can also kill gut microbiota to gain ecological niche by increasing T6SS and virulence. The free deoxycholic acid metabolized by the gut microbiota in turn inhibits the toxicity of *V. cholerae* T6SS. Therefore, gut microbiota can resist the killing effect of pathogenic bacteria by adjusting bile acid metabolism (Bachmann et al., 2015). Pathogen-susceptible mice can acquire colonization resistance to *V. cholerae* by fecal microbiota transplantation (FMT). Further research has found that *Blautia obeum*'s BSH enzyme deconjugate *tcpA* expression dependent secondary conjugated bile salts to down-regulate the expression of virulence gene in *V. cholerae* (Alavi et al., 2020). *V. cholerae* has reduced cell membrane permeability and upregulate *acrAB* gene expression to encode RND family outflow pump for avoiding accumulation toxicity of intracellular bile acids after sensing bile acid stimulation *in vitro*. This phenomenon is also found in rabbit isolated intestine models (Chatterjee et al., 2004). In addition, *V. cholerae* upregulates the porin OmpU and downregulates OmpT to resist deoxycholic acid stress via toxR (Provenzano and Klose, 2000; Ante et al., 2015). Biofilm-free *V. cholerae* activates *vps* gene and *vpsR* transcriptional activator expression to form biofilms against bile acids toxicity after deoxycholic acid and cholic acid salt stimulation (Hung et al., 2006). Thus, while the gut microbiota can de-conjugate secondary conjugated bile salts to reduce *V. cholerae* T6SS and virulence, *V. cholerae* enhances colonization by forming biofilms, upregulating the RND efflux pump family proteins, and regulating porins against bile acids toxicity.

**Figure 4:**
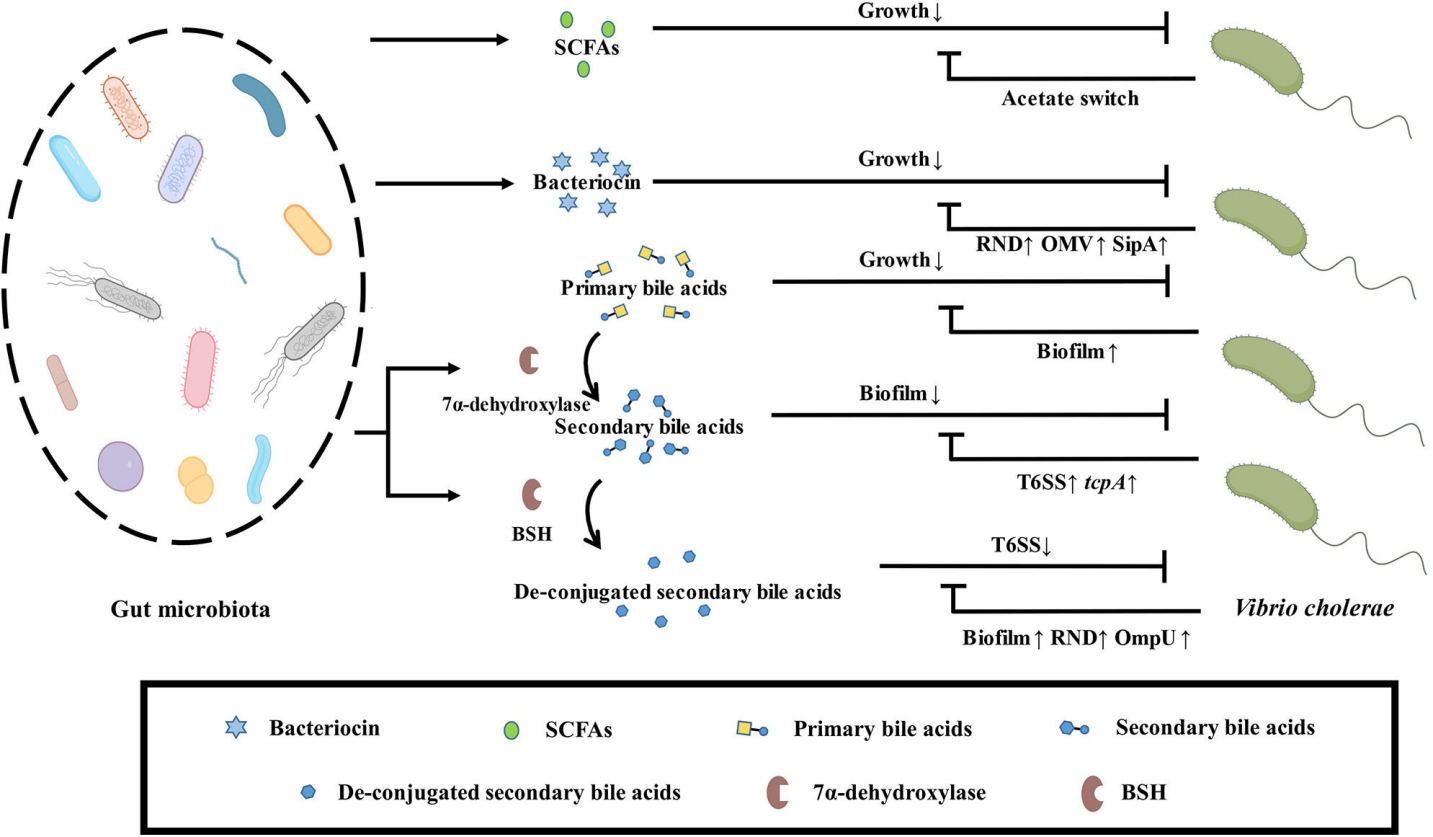
Effect of bioactive metabolites produced by gut microbiota on *V. cholerae*. SCFAs secreted by the gut microbiota inhibit the growth of *V. cholerae*, which depletes the intestinal microenvironment of acetate via acetate switch to resist this stress. Bacteriocins secreted by the gut microbiota also inhibit the growth of *V. cholerae*, which excretes bacteriocins through upregulation of the RND efflux pump family related-genes, outer membrane vesicles, and the periplasmic space protein SipA to avoid accumulating toxicity. Primary bile salts inhibit the growth of *V. cholerae*, which resists stress by forming biofilms. Secondary bile acids formed by the metabolism of primary bile salts by gut microbiota dissociate the biofilm of *V. cholerae*, but *V. cholerae* kills gut microbiota by upregulating virulence and T6SS to gain ecological niche. Gut microbiota can reduce the toxicity of T6SS by de-conjugation of secondary bile salts by BSH, but *V. cholerae* can enhance colonization by forming biofilms, upregulating the RND efflux pump family related-genes and the porin OmpU to resist stress.

Probiotic flora abundance correlates with the concentration of SCFAs in the intestinal environment, and correspondingly, SCFAs resist *V. cholerae* infection. SCFAs, the metabolites involved in the regulation of many physiological functions, and their mechanism of protecting against *V. cholerae* infection are unclear. At present, the research on the antagonism of bacteriocin produced by gut microbiota to *V. cholerae* mainly focuses on the genus *Streptococcus*, and little research has been done on the production of bacteriocins by other commensal gut bacteria. Also, the research on the molecular mechanism of *V. cholerae* in response to antimicrobial peptides mainly uses host-derived antimicrobial peptides, and the interaction mechanism between *V. cholerae* and bacteriocins is also unclear. Studies on bile acids and *V. cholerae* have focused on the effects of bile acid salts conjugation and de-conjugation on the virulence of *V. cholerae*. In addition, bile acids are metabolized by BSH enzymes from the gut microbiota and then undergo metabolic pathways such as 7α-HSDH and 3α-HSDH to produce other types of bile acids. The interaction of these kinds of bile acids with *V. cholerae* need more research (Figure 4).

## Conclusions and Perspectives

The virulence regulation network of *V. cholerae* has been well-studied. However, the classical animal model and the germ-free animal model of *V. cholerae* have ignored the role of gut microbiota. Interestingly, gut microbiota can not only use intestinal barrier to directly resist the invasion of *V. cholerae*, but also inhibit the colonization of *V. cholerae* through their metabolites, including autoinducer signaling molecules, antimicrobial peptides, short-chain fatty acids, bile salts and so on (Ducarmon et al., 2019). Correspondingly, *V. cholerae*, an intestinal pathogen, adjusts the expressions of its genes to respond to stress in the terms of T6SS, QS, ROS/pH, biofilm.

So far, the ways in which many QS signaling molecules produced by host or gut microbiota communicate with pathogens remain unknown, making it difficult to find novel quorum quenching molecules to reduce the pathogenicity of *V. cholerae*. Furthermore, traditional antibiotic therapy is facing a big challenge, which involves the drug resistance and the accelerating evolution of pathogens. Thus, the treatment of cholera by regulating the microecology of gut microbiota will be a potential therapy.

The intestine is a complex micro-ecological system, in which gut microbes have complex composition, with dynamic adjustment according to the external environment disturbance. So, the investigation of gut microbes and pathogen interactions require multidisciplinary technology platforms, including big data analysis, Next-generation sequencing, *in situ* fluorescence microscopic imagination, and lab-in-chip automatic systems (Baumler and Sperandio, 2016). Gut microbiota is the important and active component in the gastrointestinal tract, which activity should be considered in studies of the pathogenesis of enteric pathogens, representatively, *V. cholerae*. The detailed information of molecular crosstalk between commensal gut bacteria and enteric pathogens would shed a light on the prevention and control of all infectious disease.

## Author Contributions

ZL provided the general concept. ZQ, XY, GC, and ZL drafted the initial concept of manuscript, and wrote the manuscript. CP provided the critical review of the manuscript. All the authors have seen and approved the final manuscript.

## Conflict of Interest

The authors declare that the research was conducted in the absence of any commercial or financial relationships that could be construed as a potential conflict of interest.

## References

[B1] Alavi S. Mitchell J. D. Cho J. Y. Liu R. Macbeth J. C. Hsiao A. (2020). Interpersonal gut microbiome variation drives susceptibility and resistance to cholera infection. Cell 181, 1533–1546.e13. 10.1016/j.cell.2020.05.036 32631492 PMC7394201

[B2] Albert M. J. (1994). *Vibrio cholerae* O139 Bengal. J. Clin. Microbiol. 32, 2345–2349. 10.1128/JCM.32.10.2345-2349.1994 7814463 PMC264063

[B3] Alvarez A. H. Martinez Velazquez M. Prado Montes de Oca E. (2018). Human beta-defensin 1 update: potential clinical applications of the restless warrior. Int. J. Biochem. Cell. Biol. 104, 133–137. 10.1016/j.biocel.2018.09.007 30236992

[B4] Ante V. M. Bina X. R. Howard M. F. Sayeed S. Taylor D. L. Bina J. E. (2015). *Vibrio cholerae leuO* transcription is positively regulated by ToxR and contributes to bile resistance. J. Bacteriol. 197, 3499–3510. 10.1128/JB.00419-15 26303831 PMC4621094

[B5] Bachmann V. Kostiuk B. Unterweger D. Diaz-Satizabal L. Ogg S. Pukatzki S. (2015). Bile salts modulate the mucin-activated type VI secretion system of pandemic *Vibrio cholerae*. PLoS Negl. Trop. Dis. 9:e0004031. 10.1371/journal.pntd.0004031 26317760 PMC4552747

[B6] Basler M. Ho B. T. Mekalanos J. J. (2013). Tit-for-tat: type VI secretion system counterattack during bacterial cell-cell interactions. Cell 152, 884–894. 10.1016/j.cell.2013.01.042 23415234 PMC3616380

[B7] Basler M. Pilhofer M. Henderson G. P. Jensen G. J. Mekalanos J. J. (2012). Type VI secretion requires a dynamic contractile phage tail-like structure. Nature 483, 182–186. 10.1038/nature10846 22367545 PMC3527127

[B8] Baumler A. J. Sperandio V. (2016). Interactions between the microbiota and pathogenic bacteria in the gut. Nature 535, 85–93. 10.1038/nature18849 27383983 PMC5114849

[B9] Beyhan S. Bilecen K. Salama S. R. Casper-Lindley C. Yildiz F. H. (2007). Regulation of rugosity and biofilm formation in *Vibrio cholerae*: comparison of VpsT and VpsR regulons and epistasis analysis of *vpsT, vpsR*, and *hapR*. J. Bacteriol. 189, 388–402. 10.1128/JB.00981-06 17071756 PMC1797413

[B10] Bina X. R. Provenzano D. Nguyen N. Bina J. E. (2008). *Vibrio cholerae* RND family efflux systems are required for antimicrobial resistance, optimal virulence factor production, and colonization of the infant mouse small intestine. Infect. Immun. 76, 3595–3605. 10.1128/IAI.01620-07 18490456 PMC2493215

[B11] Bingle L. E. Bailey C. M. Pallen M. J. (2008). Type VI secretion: a beginner's guide. Curr. Opin. Microbiol. 11, 3–8. 10.1016/j.mib.2008.01.006 18289922

[B12] Booth S. C. Smith W. P. J. (2020). Light sheets unveil host-microorganism interactions. Nat. Rev. Microbiol. 18:65. 10.1038/s41579-019-0318-y 31873195

[B13] Broms J. E. Ishikawa T. Wai S. N. Sjostedt A. (2013). A functional VipA-VipB interaction is required for the type VI secretion system activity of *Vibrio cholerae* O1 strain A1552. BMC Microbiol. 13:96. 10.1186/1471-2180-13-96 23642157 PMC3656785

[B14] Bueno E. Sit B. Waldor M. K. Cava F. (2018). Anaerobic nitrate reduction divergently governs population expansion of the enteropathogen *Vibrio cholerae*. Nat. Microbiol. 3, 1346–1353. 10.1038/s41564-018-0253-0 30275512 PMC6443258

[B15] Cha M. K. Hong S. K. Lee D. S. Kim I. H. (2004). *Vibrio cholerae* thiol peroxidase-glutaredoxin fusion is a 2-Cys TSA/AhpC subfamily acting as a lipid hydroperoxide reductase. J. Biol. Chem. 279, 11035–11041. 10.1074/jbc.M312657200 14702341

[B16] Chairatana P. Nolan E. M. (2017). Human alpha-defensin 6: a small peptide that self-assembles and protects the host by entangling microbes. Acc. Chem. Res. 50, 960–967. 10.1021/acs.accounts.6b00653 28296382 PMC5747246

[B17] Chakraborty K. Ghosh S. Koley H. Mukhopadhyay A. K. Ramamurthy T. Saha D. R.. (2008). Bacterial exotoxins downregulate cathelicidin (hCAP-18/LL-37) and human beta-defensin 1 (HBD-1) expression in the intestinal epithelial cells. Cell. Microbiol. 10, 2520–2537. 10.1111/j.1462-5822.2008.01227.x 18717821

[B18] Chatterjee A. Chaudhuri S. Saha G. Gupta S. Chowdhury R. (2004). Effect of bile on the cell surface permeability barrier and efflux system of *Vibrio cholerae*. J. Bacteriol. 186, 6809–6814. 10.1128/JB.186.20.6809-6814.2004 15466033 PMC522216

[B19] Chen D. Wu J. Jin D. Wang B. Cao H. (2019). Fecal microbiota transplantation in cancer management: current status and perspectives. Int. J. Cancer 145, 2021–2031. 10.1002/ijc.32003 30458058 PMC6767494

[B20] Chen X. Schauder S. Potier N. Van Dorsselaer A. Pelczer I. Bassler B. L.. (2002). Structural identification of a bacterial quorum-sensing signal containing boron. Nature 415, 545–549. 10.1038/415545a 11823863

[B21] Chung L. K. Raffatellu M. (2019). G.I. Pros: Antimicrobial defense in the gastrointestinal tract. Semin. Cell. Dev. Biol. 88, 129–137. 10.1016/j.semcdb.2018.02.001 29432952 PMC6087682

[B22] Cianfanelli F. R. Alcoforado Diniz J. Guo M. De Cesare V. Trost M. Coulthurst S. J. (2016). VgrG and PAAR proteins define distinct versions of a functional type VI secretion system. PLoS Pathog. 12:e1005735. 10.1371/journal.ppat.1005735 27352036 PMC4924876

[B23] Dalile B. Van Oudenhove L. Vervliet B. Verbeke K. (2019). The role of short-chain fatty acids in microbiota-gut-brain communication. Nat. Rev. Gastroenterol. Hepatol. 16, 461–478. 10.1038/s41575-019-0157-3 31123355

[B24] Dong T. G. Ho B. T. Yoder-Himes D. R. Mekalanos J. J. (2013). Identification of T6SS-dependent effector and immunity proteins by Tn-seq in *Vibrio cholerae*. Proc. Natl. Acad. Sci. U.S.A. 110, 2623–2628. 10.1073/pnas.1222783110 23362380 PMC3574944

[B25] Duan F. March J. C. (2008). Interrupting *Vibrio cholerae* infection of human epithelial cells with engineered commensal bacterial signaling. Biotechnol. Bioeng. 101, 128–134. 10.1002/bit.21897 18433007

[B26] Duan F. March J. C. (2010). Engineered bacterial communication prevents *Vibrio cholerae* virulence in an infant mouse model. Proc. Natl. Acad. Sci. U.S.A. 107, 11260–11264. 10.1073/pnas.1001294107 20534565 PMC2895089

[B27] Ducarmon Q. R. Zwittink R. D. Hornung B. V. H. van Schaik W. Young V. B. Kuijper E. J. (2019). Gut microbiota and colonization resistance against bacterial enteric infection. Microbiol. Mol. Biol. Rev. 83:e00007-19. 10.1128/MMBR.00007-19 31167904 PMC6710460

[B28] Duperthuy M. Sjostrom A. E. Sabharwal D. Damghani F. Uhlin B. E. Wai S. N. (2013). Role of the *Vibrio cholerae* matrix protein Bap1 in cross-resistance to antimicrobial peptides. PLoS Pathog. 9:e1003620. 10.1371/journal.ppat.1003620 24098113 PMC3789753

[B29] Faruque S. M. Biswas K. Udden S. M. Ahmad Q. S. Sack D. A. Nair G. B.. (2006). Transmissibility of cholera: *in vivo*-formed biofilms and their relationship to infectivity and persistence in the environment. Proc. Natl. Acad. Sci. U.S.A. 103, 6350–6355. 10.1073/pnas.0601277103 16601099 PMC1458881

[B30] Fast D. Kostiuk B. Foley E. Pukatzki S. (2018). Commensal pathogen competition impacts host viability. Proc. Natl. Acad. Sci. U.S.A. 115, 7099–7104. 10.1073/pnas.1802165115 29915049 PMC6142279

[B31] Fast D. Petkau K. Ferguson M. Shin M. Galenza A. Kostiuk B.. (2020). *Vibrio cholerae*-symbiont interactions inhibit intestinal repair in *drosophila*. Cell Rep. 30, 1088–1100.e1085. 10.1016/j.celrep.2019.12.094 31995751 PMC9684019

[B32] Fu Y. Ho B. T. Mekalanos J. J. (2018). Tracking *Vibrio cholerae* cell-cell interactions during infection reveals bacterial population dynamics within intestinal microenvironments. Cell Host Microbe 23, 274–281.e272. 10.1016/j.chom.2017.12.006 29398650 PMC6031135

[B33] Fu Y. Waldor M. K. Mekalanos J. J. (2013). Tn-Seq analysis of *Vibrio cholerae* intestinal colonization reveals a role for T6SS-mediated antibacterial activity in the host. Cell Host Microbe 14, 652–663. 10.1016/j.chom.2013.11.001 24331463 PMC3951154

[B34] Gorelik O. Levy N. Shaulov L. Yegodayev K. Meijler M. M. Sal-Man N. (2019). *Vibrio cholerae* autoinducer-1 enhances the virulence of enteropathogenic *Escherichia coli*. Sci. Rep. 9:4122. 10.1038/s41598-019-40859-1 30858454 PMC6411865

[B35] Hammami R. Fernandez B. Lacroix C. Fliss I. (2013). Anti-infective properties of bacteriocins: an update. Cell. Mol. Life Sci. 70, 2947–2967. 10.1007/s00018-012-1202-3 23109101 PMC11113238

[B36] Hammer B. K. Bassler B. L. (2003). Quorum sensing controls biofilm formation in *Vibrio cholerae*. Mol. Microbiol 50, 101–104. 10.1046/j.1365-2958.2003.03688.x 14507367

[B37] Hang S. Purdy A. E. Robins W. P. Wang Z. Mandal M. Chang S.. (2014). The acetate switch of an intestinal pathogen disrupts host insulin signaling and lipid metabolism. Cell Host Microbe. 16, 592–604. 10.1016/j.chom.2014.10.006 25525791 PMC4272434

[B38] Hawver L. A. Giulietti J. M. Baleja J. D. Ng W. L. (2016). Quorum sensing coordinates cooperative expression of pyruvate metabolism genes to maintain a sustainable environment for population stability. MBio 7:16. 10.1128/mBio.01863-16 27923919 PMC5142617

[B39] Hay A. J. Zhu J. (2015). Host intestinal signal-promoted biofilm dispersal induces *Vibrio cholerae* colonization. Infect. Immun. 83, 317–323. 10.1128/IAI.02617-14 25368110 PMC4288906

[B40] Herzog R. Peschek N. Fröhlich K. S. Schumacher K. Papenfort K. (2019). Three autoinducer molecules act in concert to control virulence gene expression in *Vibrio cholerae*. Nucleic Acids Res. 47, 3171–3183. 10.1093/nar/gky1320 30649554 PMC6451090

[B41] Higgins D. A. Pomianek M. E. Kraml C. M. Taylor R. K. Semmelhack M. F. Bassler B. L. (2007). The major *Vibrio cholerae* autoinducer and its role in virulence factor production. Nature 450, 883–886. 10.1038/nature06284 18004304

[B42] Holowko M. B. Wang H. Jayaraman P. Poh C. L. (2016). Biosensing *Vibrio cholerae* with genetically engineered *Escherichia coli*. ACS Synth. Biol 5, 1275–1283. 10.1021/acssynbio.6b00079 27529184

[B43] Hsiao A. Ahmed A. M. Subramanian S. Griffin N. W. Drewry L. L. Petri W. A.. (2014). Members of the human gut microbiota involved in recovery from *Vibrio cholerae* infection. Nature 515, 423–426. 10.1038/nature13738 25231861 PMC4353411

[B44] Hung D. T. Zhu J. Sturtevant D. Mekalanos J. J. (2006). Bile acids stimulate biofilm formation in *Vibrio cholerae*. Mol. Microbiol. 59, 193–201. 10.1111/j.1365-2958.2005.04846.x 16359328

[B45] Iatsenko I. Boquete J. P. Lemaitre B. (2018). Microbiota-derived lactate activates production of reactive oxygen species by the intestinal NADPH oxidase nox and shortens *drosophila* lifespan. Immunity 49, 929–942.e925. 10.1016/j.immuni.2018.09.017 30446385

[B46] Jemielita M. Wingreen N. S. Bassler B. L. (2018). Quorum sensing controls *Vibrio cholerae* multicellular aggregate formation. Elife 7:e42057. 10.7554/eLife.42057 30582742 PMC6351105

[B47] Kaper J. B. Morris J. G. Jr. Levine M. M. (1995). Cholera. Clin. Microbiol. Rev. 8, 48–86. 10.1128/CMR.8.1.48 7704895 PMC172849

[B48] Kaur S. Sharma P. Kalia N. Singh J. Kaur S. (2018). Anti-biofilm properties of the fecal probiotic lactobacilli against *vibrio* spp. Front. Cell. Infect. Microbiol 8:120. 10.3389/fcimb.2018.00120 29740541 PMC5928150

[B49] Kelly R. C. Bolitho M. E. Higgins D. A. Lu W. Ng W. L. Jeffrey P. D.. (2009). The *Vibrio cholerae* quorum-sensing autoinducer CAI-1: analysis of the biosynthetic enzyme CqsA. Nat. Chem. Biol 5, 891–895. 10.1038/nchembio.237 19838203 PMC2847429

[B50] Kim E. K. Lee K. A. Hyeon D. Y. Kyung M. Jun K. Y. Seo S. H.. (2020). Bacterial nucleoside catabolism controls quorum sensing and commensal-to-pathogen transition in the *drosophila* gut. Cell Host Microbe. 27, 345–357.e346. 10.1016/j.chom.2020.01.025 32078802

[B51] Kovacikova G. Lin W. Skorupski K. (2010). The LysR-type virulence activator AphB regulates the expression of genes in *Vibrio cholerae* in response to low pH and anaerobiosis. J. Bacteriol. 192, 4181–4191. 10.1128/JB.00193-10 20562308 PMC2916415

[B52] Kunkle D. E. Bina X. R. Bina J. E. (2020). *Vibrio cholerae* OmpR contributes to virulence repression and fitness at alkaline pH. Infect. Immun 88:e00141–20. 10.1128/IAI.00141-20 32284367 PMC7240085

[B53] Lee D. Kim E. J. Baek Y. Lee J. Yoon Y. Nair G. B.. (2020). Alterations in glucose metabolism in *Vibrio cholerae* serogroup O1 El Tor biotype strains. Sci. Rep. 10:308. 10.1038/s41598-019-57093-4 31941909 PMC6962216

[B54] Liu Z. Wang H. Zhou Z. Sheng Y. Naseer N. Kan B.. (2016). Thiol-based switch mechanism of virulence regulator AphB modulates oxidative stress response in *Vibrio cholerae*. Mol. Microbiol. 102, 939–949. 10.1111/mmi.13524 27625149 PMC5123930

[B55] Liu Z. Yang M. Peterfreund G. L. Tsou A. M. Selamoglu N. Daldal F.. (2011). *Vibrio cholerae* anaerobic induction of virulence gene expression is controlled by thiol-based switches of virulence regulator AphB. Proc. Natl. Acad. Sci. U.S.A. 108, 810–815. 10.1073/pnas.1014640108 21187377 PMC3021084

[B56] Lo Scrudato M. Blokesch M. (2012). The regulatory network of natural competence and transformation of *Vibrio cholerae*. PLoS Genet. 8:e1002778. 10.1371/journal.pgen.1002778 22737089 PMC3380833

[B57] Logan S. L. Thomas J. Yan J. Baker R. P. Shields D. S. Xavier J. B.. (2018). The *Vibrio cholerae* type VI secretion system can modulate host intestinal mechanics to displace gut bacterial symbionts. Proc. Natl. Acad. Sci. U.S.A. 115, E3779–E3787. 10.1073/pnas.1720133115 29610339 PMC5910850

[B58] Mao N. Cubillos-Ruiz A. Cameron D. E. Collins J. J. (2018). Probiotic strains detect and suppress cholera in mice. Sci. Transl. Med. 10:eaao2586. 10.1126/scitranslmed.aao2586 29899022 PMC7821980

[B59] Meister A. Anderson M. E. (1983). Glutathione. Annu. Rev. Biochem. 52, 711–760. 10.1146/annurev.bi.52.070183.003431 6137189

[B60] Miller M. B. Skorupski K. Lenz D. H. Taylor R. K. Bassler B. L. (2002). Parallel quorum sensing systems converge to regulate virulence in *Vibrio cholerae*. Cell 110, 303–314. 10.1016/S0092-8674(02)00829-2 12176318

[B61] Miyata S. T. Kitaoka M. Brooks T. M. McAuley S. B. Pukatzki S. (2011). *Vibrio cholerae* requires the type VI secretion system virulence factor VasX to kill Dictyostelium discoideum. Infect. Immun. 79, 2941–2949. 10.1128/IAI.01266-10 21555399 PMC3191968

[B62] Monira S. Hoq M. M. Chowdhury A. K. Suau A. Magne F. Endtz H. P.. (2010). Short-chain fatty acids and commensal microbiota in the faeces of severely malnourished children with cholera rehydrated with three different carbohydrates. Eur. J. Clin. Nutr. 64, 1116–1124. 10.1038/ejcn.2010.123 20683462

[B63] Morales-Estrada A. I. Lopez-Merino A. Gutierrez-Mendez N. Ruiz E. A. Contreras-Rodriguez A. (2016). Partial characterization of bacteriocin produced by halotolerant *pediococcus acidilactici* strain QC38 isolated from traditional cotija Cheese. Pol. J. Microbiol. 65, 279–285. 10.5604/17331331.1215607 29334047

[B64] Mottawea W. Chiang C. K. Mühlbauer M. Starr A. E. Butcher J. Abujamel T.. (2016). Altered intestinal microbiota-host mitochondria crosstalk in new onset Crohn's disease. Nat. Commun. 7:13419. 10.1038/ncomms13419 27876802 PMC5122959

[B65] Nag D. Breen P. Raychaudhuri S. Withey J. H. (2018). glucose metabolism by *Escherichia coli* inhibits *vibrio cholerae* intestinal colonization of zebrafish. Infect. Immun. 86:18. 10.1128/IAI.00486-18 30249745 PMC6246912

[B66] Olasupo N. A. Olukoya D. K. Odunfa S. A. (1995). Studies on bacteriocinogenic *Lactobacillus* isolates from selected Nigerian fermented foods. J. Basic Microbiol. 35, 319–324. 10.1002/jobm.3620350507 8568643

[B67] Papenfort K. Silpe J. E. Schramma K. R. Cong J. P. Seyedsayamdost M. R. Bassler B. L. (2017). A *Vibrio cholerae* autoinducer-receptor pair that controls biofilm formation. Nat. Chem. Biol. 13, 551–557. 10.1038/nchembio.2336 28319101 PMC5391282

[B68] Parker C. T. Sperandio V. (2009). Cell-to-cell signalling during pathogenesis. Cell. Microbiol. 11, 363–369. 10.1111/j.1462-5822.2008.01272.x 19068097 PMC2786497

[B69] Provenzano D. Klose K. E. (2000). Altered expression of the ToxR-regulated porins OmpU and OmpT diminishes *Vibrio cholerae* bile resistance, virulence factor expression, and intestinal colonization. Proc. Natl. Acad. Sci. U.S.A. 97, 10220–10224. 10.1073/pnas.170219997 10944196 PMC27820

[B70] Pukatzki S. Ma A. T. Revel A. T. Sturtevant D. Mekalanos J. J. (2007). Type VI secretion system translocates a phage tail spike-like protein into target cells where it cross-links actin. Proc. Natl. Acad. Sci. U.S.A. 104, 15508–15513. 10.1073/pnas.0706532104 17873062 PMC2000545

[B71] Pukatzki S. Ma A. T. Sturtevant D. Krastins B. Sarracino D. Nelson W. C.. (2006). Identification of a conserved bacterial protein secretion system in *Vibrio cholerae* using the *Dictyostelium* host model system. Proc. Natl. Acad. Sci. U.S.A. 103, 1528–1533. 10.1073/pnas.0510322103 16432199 PMC1345711

[B72] Qadri F. Bhuiyan T. R. Dutta K. K. Raqib R. Alam M. S. Alam N. H.. (2004). Acute dehydrating disease caused by *Vibrio cholerae* serogroups O1 and O139 induce increases in innate cells and inflammatory mediators at the mucosal surface of the gut. Gut 53, 62–69. 10.1136/gut.53.1.62 14684578 PMC1773936

[B73] Quinn M. J. Resch C. T. Sun J. Lind E. J. Dibrov P. Hase C. C. (2012). NhaP1 is a K+(Na+)/H+ antiporter required for growth and internal pH homeostasis of *Vibrio cholerae* at low extracellular pH. Microbiology 158, 1094–1105. 10.1099/mic.0.056119-0 22241048 PMC3949420

[B74] Ridlon J. M. Harris S. C. Bhowmik S. Kang D. J. Hylemon P. B. (2016). Consequences of bile salt biotransformations by intestinal bacteria. Gut Microbes 7, 22–39. 10.1080/19490976.2015.1127483 26939849 PMC4856454

[B75] Ritchie J. M. Waldor M. K. (2009). *Vibrio cholerae* interactions with the gastrointestinal tract: lessons from animal studies. Curr. Top. Microbiol. Immunol. 337, 37–59. 10.1007/978-3-642-01846-6_2 19812979

[B76] Rivera-Chavez F. Mekalanos J. J. (2019). Cholera toxin promotes pathogen acquisition of host-derived nutrients. Nature 572, 244–248. 10.1038/s41586-019-1453-3 31367037 PMC6727848

[B77] Rowe-Magnus D. A. Kao A. Y. Prieto A. C. Pu M. Kao C. (2019). Cathelicidin peptides restrict bacterial growth via membrane perturbation and induction of reactive oxygen species. MBio 10:19. 10.1128/mBio.02021-19 31506312 PMC6737244

[B78] Russell A. B. Hood R. D. Bui N. K. LeRoux M. Vollmer W. Mougous J. D. (2011). Type VI secretion delivers bacteriolytic effectors to target cells. Nature 475, 343–347. 10.1038/nature10244 21776080 PMC3146020

[B79] Russell A. B. LeRoux M. Hathazi K. Agnello D. M. Ishikawa T. Wiggins P. A.. (2013). Diverse type VI secretion phospholipases are functionally plastic antibacterial effectors. Nature 496, 508–512. 10.1038/nature12074 23552891 PMC3652678

[B80] Saul-McBeth J. Matson J. S. (2019). A periplasmic antimicrobial peptide-binding protein is required for stress survival in *vibrio cholerae*. Front. Microbiol. 10:161. 10.3389/fmicb.2019.00161 30804918 PMC6370654

[B81] Schauder S. Shokat K. Surette M. G. Bassler B. L. (2001). The LuxS family of bacterial autoinducers: biosynthesis of a novel quorum-sensing signal molecule. Mol. Microbiol. 41, 463–476. 10.1046/j.1365-2958.2001.02532.x 11489131

[B82] Schwarz S. West T. E. Boyer F. Chiang W. C. Carl M. A. Hood R. D.. (2010). *Burkholderia* type VI secretion systems have distinct roles in eukaryotic and bacterial cell interactions. PLoS Pathog. 6:e1001068. 10.1371/journal.ppat.1001068 20865170 PMC2928800

[B83] Sengupta C. Ekka M. Arora S. Dhaware P. D. Chowdhury R. Raychaudhuri S. (2017). Cross feeding of glucose metabolism byproducts of *Escherichia coli* human gut isolates and probiotic strains affect survival of *Vibrio cholerae*. Gut Pathog. 9:3. 10.1186/s13099-016-0153-x 28105081 PMC5240293

[B84] Sengupta C. Mukherjee O. Chowdhury R. (2016). Adherence to Intestinal Cells Promotes Biofilm Formation in *Vibrio cholerae*. J. Infect. Dis. 214, 1571–1578. 10.1093/infdis/jiw435 27638940

[B85] Shao Y. Bassler B. L. (2014). Quorum regulatory small RNAs repress type VI secretion in *Vibrio cholerae*. Mol. Microbiol. 92, 921–930. 10.1111/mmi.12599 24698180 PMC4038675

[B86] Shikuma N. J. Fong J. C. Odell L. S. Perchuk B. S. Laub M. T. Yildiz F. H. (2009). Overexpression of VpsS, a hybrid sensor kinase, enhances biofilm formation in *Vibrio cholerae*. J. Bacteriol. 191, 5147–5158. 10.1128/JB.00401-09 19525342 PMC2725581

[B87] Shirin T. Rahman A. Danielsson A. Uddin T. Bhuyian T. R. Sheikh A.. (2011). Antimicrobial peptides in the duodenum at the acute and convalescent stages in patients with diarrhea due to *Vibrio cholerae* O1 or enterotoxigenic *Escherichia coli* infection. Microbes. Infect. 13, 1111–1120. 10.1016/j.micinf.2011.06.014 21782033

[B88] Shneider M. M. Buth S. A. Ho B. T. Basler M. Mekalanos J. J. Leiman P. G. (2013). PAAR-repeat proteins sharpen and diversify the type VI secretion system spike. Nature 500, 350–353. 10.1038/nature12453 23925114 PMC3792578

[B89] Spelhaug S. R. Harlander S. K. (1989). Inhibition of foodborne bacterial pathogens by bacteriocins from *Lactococcus lactis* and *Pediococcus pentosaceous* (1). J. Food Prot. 52, 856–862. 10.4315/0362-028X-52.12.856 31003362

[B90] Suckow G. Seitz P. Blokesch M. (2011). Quorum sensing contributes to natural transformation of *Vibrio cholerae* in a species-specific manner. J. Bacteriol. 193, 4914–4924. 10.1128/JB.05396-11 21784943 PMC3165701

[B91] Theriot C. M. Petri W. A. Jr. (2020). Role of microbiota-derived bile acids in enteric infections. Cell 181, 1452–1454. 10.1016/j.cell.2020.05.033 32589955 PMC8162987

[B92] Toska J. Ho B. T. Mekalanos J. J. (2018). Exopolysaccharide protects *Vibrio cholerae* from exogenous attacks by the type 6 secretion system. Proc. Natl. Acad. Sci. U.S.A. 115, 7997–8002. 10.1073/pnas.1808469115 30021850 PMC6077691

[B93] Wahlström A. Sayin S. I. Marschall H. U. Bäckhed F. (2016). Intestinal crosstalk between bile acids and microbiota and its impact on host metabolism. Cell Metab. 24, 41–50. 10.1016/j.cmet.2016.05.005 27320064

[B94] Wang H. Naseer N. Chen Y. Zhu A. Y. Kuai X. Galagedera N.. (2017). OxyR2 Modulates OxyR1 activity and *Vibrio cholerae* oxidative stress response. Infect. Immun. 85:e00929–16. 10.1128/IAI.00929-16 28138024 PMC5364302

[B95] Wang H. Xing X. Wang J. Pang B. Liu M. Larios-Valencia J.. (2018). Hypermutation-induced *in vivo* oxidative stress resistance enhances *Vibrio cholerae* host adaptation. PLoS Pathog 14:e1007413. 10.1371/journal.ppat.1007413 30376582 PMC6226196

[B96] Watve S. Barrasso K. Jung S. A. Davis K. J. Hawver L. A. Khataokar A.. (2020). Parallel quorum-sensing system in *Vibrio cholerae* prevents signal interference inside the host. PLoS Pathog 16:e1008313. 10.1371/journal.ppat.1008313 32059031 PMC7046293

[B97] Xia X. Larios-Valencia J. Liu Z. Xiang F. Kan B. Wang H.. (2017). OxyR-activated expression of Dps is important for *Vibrio cholerae* oxidative stress resistance and pathogenesis. PLoS ONE 12:e0171201. 10.1371/journal.pone.0171201 28151956 PMC5289545

[B98] Yang M. Liu Z. Hughes C. Stern A. M. Wang H. Zhong Z.. (2013). Bile salt-induced intermolecular disulfide bond formation activates *Vibrio cholerae* virulence. Proc. Natl. Acad. Sci. U.S.A. 110, 2348–2353. 10.1073/pnas.1218039110 23341592 PMC3568309

[B99] Yardeni T. Tanes C. E. Bittinger K. Mattei L. M. Schaefer P. M. Singh L. N.. (2019). Host mitochondria influence gut microbiome diversity: A role for ROS. Sci. Signal 12:aaw3159. 10.1126/scisignal.aaw3159 31266851

[B100] Yoon M. Y. Min K. B. Lee K. M. Yoon Y. Kim Y. Oh Y. T.. (2016). A single gene of a commensal microbe affects host susceptibility to enteric infection. Nat. Commun. 7:11606. 10.1038/ncomms11606 27173141 PMC5482719

[B101] Yoon S. H. Waters C. M. (2019). *Vibrio cholerae*. Trends Microbiol. 27, 806–807. 10.1016/j.tim.2019.03.005 31029488 PMC6713289

[B102] You J. S. Yong J. H. Kim G. H. Moon S. Nam K. T. Ryu J. H.. (2019). Commensal-derived metabolites govern *Vibrio cholerae* pathogenesis in host intestine. Microbiome 7:132. 10.1186/s40168-019-0746-y 31521198 PMC6744661

[B103] Zhao W. Caro F. Robins W. Mekalanos J. J. (2018). Antagonism toward the intestinal microbiota and its effect on *Vibrio cholerae* virulence. Science 359, 210–213. 10.1126/science.aap8775 29326272 PMC8010019

[B104] Zhu J. Miller M. B. Vance R. E. Dziejman M. Bassler B. L. Mekalanos J. J. (2002). Quorum-sensing regulators control virulence gene expression in *Vibrio cholerae*. Proc. Natl. Acad. Sci. U.S.A. 99, 3129–3134. 10.1073/pnas.052694299 11854465 PMC122484

